# Establishing facility reference levels and comparing to international diagnostic reference levels in paediatric general X-ray

**DOI:** 10.1007/s13246-025-01563-9

**Published:** 2025-05-30

**Authors:** Samuel M. Lilli, Amanda A. Perdomo

**Affiliations:** Department of Medical Imaging, The Royal Children’s Hospital Melbourne, 50 Flemington Road, Parkville, VIC 3052 Australia

**Keywords:** Diagnostic reference levels, Paediatric, Radiography

## Abstract

Diagnostic Reference Levels (DRLs) can be used to assess the radiation exposure for specific protocols and identify areas of potential optimisation. Despite paediatric general X-ray (GXR) being a relatively low dose modality, due to the high radiosensitivity of children, it is imperative that doses remain as low as reasonably achievable (ALARA). This study aims to compare our institute’s Dose-Area-Product (DAP) to currently available local, national, and regional DRLs, as there are currently no Australian DRL values established for paediatric GXR. The DAPs for GXR protocols are recorded in a commercially available DMS software, MyXrayDose, which generates a report of the Facility Reference Levels (FRLs) for all GXR protocols. As MyXrayDose uses age categories, our FRLs were converted to weight-based FRLs using the 50th percentile values derived from the World Health Organisation and Centres for Disease Control Weight-for-age charts. These were compared to published DRLs from eleven different countries and regions of the world. Pelvis Anterior-Posterior (AP)/Posterior-Anterior (PA), Abdomen AP/PA, Thorax AP/PA and Thorax lateral protocols were compared to available national and regional DRLs. For example, from 1st July 2023–30th June 2024, 1008 Abdomen AP/PA X-rays were conducted in Room 1 with a fixed GXR unit. The FRL for 31.2–56.5 kg (10–15 years) patients in Room 1 (1093 mGy.cm^2^) was more than 2.3 times greater than the European DRL (475 mGy.cm^2^). The FRLs for patients with a mean weight of 6 kg and 14 kg were below the European DRL whilst 25 kg, 44 kg and 60 kg patients exceeded the European Abdomen AP/PA DRL. The establishment of DRLs helps institutes identify potential areas of optimisation. As some of our GXR protocols exceed the European DRLs, the next step at our institute is to complete a multi-disciplinary image quality assessment to identify if it is possible to optimise these protocols.

## Introduction

Diagnostic imaging protocols, utilising ionising radiation, provide information about patient anatomy which can help determine patient management. However, it is well documented that paediatric patients exposed to ionising radiation are the most at risk of stochastic radiation effects within the population [1]. One contributing modality is general X-ray (GXR) which uses a relatively low amount of radiation per examination compared to other diagnostic imaging modalities. However, as per the Australian Bureau of Statistics, GXR is the most commonly used ionising radiation modality [2]. This may lead to a sizeable population dose, with an increased radiation exposure resulting in an increased probability of stochastic radiation effects.

Diagnostic Reference Levels (DRLs) were first proposed by the International Commission for Radiological Protection (ICRP) in 1996 as a method to promote optimisation of radiation protection of patients [3]. Facilities participating in a DRL survey will submit protocol, age classification, and dose information data for each protocol. A facility reference level (FRL), calculated as the 50th percentile (median) of that facility’s data for each unit can then be generated. The DRL is determined as the 75th percentile of the resulting FRL distribution from participating facilities, whilst the Local DRL (LDRL) is the 75th percentile of the FRL distribution within a facility. This DRL value is an indicative measure used to assess whether, in routine conditions, the amount of radiation used is unusually high (or low) for a specified protocol. If a DRL is exceeded, a review of that protocol should be completed, to identify any areas of potential optimisation [4].

Despite DRLs promoting the optimisation of diagnostic imaging protocols, it inherently holds some issues. For example, in their current form, DRLs may be misused to describe the radiation dose an individual receives. They can also be inappropriately applied as the radiation dose limit of a protocol, however dose limits do not apply to the medical exposure of patients [5]. DRL collection surveys can also be inaccurate due to ‘cherry-picking’ of data and can misrepresent the patient demographic of an institute. Data used in DRL surveys are to be from patient examinations only and phantom studies are considered to be inappropriate as there are no defined clinical objectives, and staff may respond differently to a controlled examination [4]. The dose cannot be the sole consideration, and it is crucial that achieving a suitable image quality to answer the clinical question remains the primary objective.

In Australia, a national DRL survey is facilitated by the Australian Radiation Protection and Nuclear Safety Agency (ARPANSA). Currently in Australia there are paediatric DRLs established for Multi Detector Computed Tomography (MDCT), however there are no paediatric GXR DRLs, resulting in a missed opportunity for optimisation in paediatric radiography across Australia. This may be attributed to the lower imaging rate of paediatric patients, the small radiation dose per examination in GXR, and the substantial number of protocols available.

In 2016, the European DRLs for Paediatric Imaging (PiDRL) guidelines were established [6, 7]. These guidelines recommend the use of weight-based patient groupings for all body protocols. The current paediatric MDCT DRLs in Australia utilise the following age-based groupings; 0–4 years, 5–14 years and 15 years and older [8]. This presents issues when considering a 0-year-old female in the 5th weight percentile of 2.5 kg is in the same age category as a 4-year-old male patient in the 95th weight percentile of 20.4 kg [9]. This weight factor of 8.2 is double what you may expect to see in an adult cohort (40–160 kg, factor of 4) [10]. Weight variation is particularly pertinent in tertiary paediatric institutions, such as ours, where many patients are not in a ‘healthy’ weight category due to underlying complex medical issues. To overcome this issue the European Commission have proposed the following weight groupings (Table 1) which have also been endorsed by the ICRP.

**Table 1:** Weight groupings suggested by European guidelines [7]

Description	Weight group (kg)	Age group based on weight-for-age charts	Most common age groups used for the previous national DRLs (years)
Neonate	< 5	< 1 month	0
Infant, toddler, and early childhood	5-<15	1 month to < 4 years	1
Middle childhood	15-<30	4-<10 years	5
Early adolescence	30-<50	10-<14 years	10
Late adolescence	50-<80	14-<18 years	15

Note these weight categories are not recommended for head protocols as head size varies less with age than body size [4]. Therefore head protocols have been excluded

Due to low examination numbers, an alternative approach is to generate a weight based DRL curve which is a mathematical fit based on a dose indicator as a function of patient weight [11]. For each protocol, the median weight and third quartile of the dose indicator is plotted with an exponential line of best fit using the form:

$$\:D\left(w\right)={D}_{q3}\bullet\:{e}^{\left(k\bullet\:w\right)}$$

Where D(w) is the DRL for a particular dose indicator for each patient weight (w). The exponential growth is indicated by a derived regression coefficient (k), with D_q3_ indicating the third quartile value of the median weight from your survey data. This exponential increase in dose is based on the exponential increase in patient attenuation with increasing size [11]. A variation of this method has been used in Finland to derive national paediatric DRLs based on patient thickness as a substitute for weight [12]. This DRL curve helps overcome the issue of poor statistics associated with using discrete weight categories, in particular for small sample sizes, and produces a line of best fit using patient weight and a dose indicator.

The ICRP recommend the primary DRL quantity for GXR as the Dose-Area-Product (DAP) [4, 7]. This quantity is usually available on fixed imaging units for centres wishing to partake in DRL surveys and considers the full radiation exposure of the patient.

The primary aim of this study is to benchmark our institutes GXR exposures against available paediatric GXR DRL data (local, national, regional) using DAP as the dose indicator. This will then be used to assess whether GXR protocols at our institute may require review. This is to ensure our doses are As Low As Reasonably Achievable (ALARA) whilst maintaining an acceptable level of image quality.

## Methods

A review of available local, national, and regional DRLs was conducted to find benchmark data. As there is no available Australian DRL, international sources were required. The only available comparison found within Australia is presented in Agarwal and Newbery, where mean DAPs from radiology practices in Tasmania have been reported [13].

Available data categorised based on age was converted to weight-based categories. The 50th percentile weight-for-age data published by the World Health Organization (WHO) was used to convert patient age groupings to weight groupings [9]. This data is only available for patients up to the age of ten. Reference data beyond the age of 10 years and up to 18 years is available through the Centres for Disease Control and Prevention (CDC), with the 50th weight percentile in these age groups used [20].

The DAP for the three fixed and one resus GXR unit at The Royal Children’s Hospital Melbourne (RCH) were extracted from the dose management software (DMS), MyXrayDose [21]. Mobile X-ray examinations were excluded from this work as not all units contain a DAP meter to record patient DAP data. The GXR units (Room 1, Room 3, Room 4, Resus) use indirect digital detector technology (Shimadzu RADSpeed Pro UD 150B-10 with Canon CXDI-70 C CSI(CsI: Tl) wireless digital detector) installed in 2011.

Four commonly performed GXR protocols were assessed; Pelvis Anterior-Posterior (AP)/Posterior-Anterior (PA) X-ray, Thorax AP/PA X-ray, Thorax Lateral X-ray, and Abdomen AP/PA X-ray. Using MyXrayDose, a ‘Reference Dose Review’ was generated with patient data from 1st July 2023–30th June 2024. The report identifies the patient age grouping, the study type, the median DAP for a study type, and the number of samples. MyXrayDose produces an FRL value which is taken as the median DAP for each age group and study type. RCH patient age data was converted to weight categories using the 50th percentile weight-for-age data from WHO and CDC [9, 20]. The data was exported to Microsoft Excel, where data was categorised by weight. Patient age-for-weight and derived mean weights are shown in Table 2, with the mean weights rounded to the nearest kilogram. In smaller weight categories, outliers are numerically less impactful on the mean weight compared to larger weight categories. However, as patient age increases there are an increased number of outliers due to the presentation of clinical conditions. However, as there are expected to be outliers on both sides of the weight ranges listed in Table 2 (due to varying clinical conditions i.e., achondroplasia, obesity) the mean and mediant weight should be approximately equal.

**Table 2:** Patient age-for-weight and mean weight [9, 20, 21]

MyXrayDose Age Categories	WHO and CDC 50th percentile weight-for-age (kg)	Mean Weight (kg)
Newborn	3.2–9.6	6
1–5 years	8.9–18.3	14
5–10 years	18.2–31.9	25
10–15 years	31.2–56.5	44
Adult	52.1–67.3	60

The resulting median dose indicator for each study type and weight category, were compared to available DRL values. For example, if a 6 kg patient received a Thorax AP/PA examination, what DAP would they receive at RCH compared to all available data for newborn Thorax AP/PA examinations.

This project has been assessed as exempt from Human Research Ethics Committee review by the RCH Research Ethics and Governance Office (Project Title: 4006).

## Results

Table 3 displays the list of all available local, national, and regional DRLs found during the review, which had paediatric GXR measurements using DAP as the dose indicator. Head protocols have not been included in this study, as weight based DRLs are not recommended for head protocols [4]. In addition, skull X-rays often have a lower diagnosis rate when compared to CT in children with skull fractures [14], therefore the rate of skull X-rays are reduced. Studies and DRLs using air kerma (K_a, r_) only have been excluded. An example of this is the Japanese National DRL [15], which is set using K_a, r_.

**Table 3:** Available local, National, and regional DRLs

Country/Region	Exam	Collection Period	Number of Participating sites
Austria [16]	Thorax (AP/PA) Abdomen (AP/PA)	September 2006 – September 2007	
Germany [7]	Thorax (AP/PA) Thorax (Lateral) Pelvis (AP/PA) Abdomen (AP/PA)	2006–2009	All German institutes
Spain [7, 17]	Thorax (AP/PA) Pelvis (AP/PA) Abdomen (AP/PA)	2011–2013	5–10% of paediatric institutes
Finland [12]	Thorax (AP/PA) Thorax (Lateral)	2004–2005	
Lithuania [7]	Thorax (AP/PA) Abdomen (AP/PA)	2009–2012	
Belgium [7]	Thorax (AP/PA) Thorax (PA + Lateral) Abdomen (AP/PA)	Not provided	17
France [18]	Thorax (AP/PA) Thorax (Lateral) Pelvis (AP/PA) Abdomen (AP/PA)	2004–2008	
Netherlands [7]	Thorax (AP/PA) Abdomen (AP/PA)	Not provided	
Europe [7]	Thorax (AP/PA) – Austria, Belgium, Germany, Spain, Finland, France, Netherlands, Lithuania Abdomen (AP/PA) – Austria, Belgium, Germany, Spain, France, Lithuania, Lithuania Pelvis (AP/PA) – Germany, France, Spain	NA (Collation of other studies)	4–8 countries 3–7 countries 2–3 countries
Ireland [19]	Pelvis (AP/PA) Thorax (AP/PA) Abdomen (AP/PA)	November 2020	7–19 14–27 8–18
Nordic (Denmark, Iceland, Norway, Sweden) [10]	Pelvis (AP/PA) Thorax (AP/PA) Abdomen (AP/PA)	2018–2019	15 13–17 18

In total, 53,681 GXR examinations were conducted at our institute from 1st July 2023–30th June 2024. Table 4 lists assessed protocols, with the mean weight, the FRL for the protocol, and the LDRL for the protocol. The available DRLs for which this patient weight corresponds to is included in Table 6. The median DAP for a 44 kg patient receiving a Pelvis AP/PA protocol at our institute in Room 1 is 1295 mGy.cm^2^, 4.2 times more than the established European DRL.

**Table 4:** RCH FRL and LDRL values found in this study. Cells **bolded and italicised** indicate that the resultant FRL or LDRL exceeds the European DRL or; if it is not available, the highest available National DRL

MyXrayDose Age Categories	Mean Weight [kg]	FRL [mGy.cm^2^] (Sample size)				LDRL [mGy.cm^2^] (Rounded to the nearest integer)
		Room 1	Room 3	Room 4	Resus	
Pelvis AP/PA						
Newborn	6	32 (74)	35 (93)	30 (54)	32 (2)	33
1–5 years	14	59 (225)	60 (265)	53 (177)	44 (10)	60
5–10 years	25	***566*** (310)	***409*** (327)	***404*** (224)	***531*** (17)	***540***
10–15 years	44	***1295*** (372)	***1158*** (344)	***1271*** (214)	***1758*** (33)	***1411***
Adult	60	***1401*** (122)	***1633*** (123)	***1869*** (87)	***1908*** (4)	***1879***
Thorax AP/PA						
Newborn	6	12 (775)	10 (118)	12 (74)	12 (11)	12
1–5 years	14	19 (1647)	19 (276)	19 (174)	19 (50)	19
5–10 years	25	35 (1340)	32 (254)	33 (142)	***66*** *(53)*	***43***
10–15 years	44	***95*** (1249)	68 (230)	***88*** (134)	***121*** (91)	***102***
Adult	60	***127*** (493)	***129*** (128)	***180*** (64)	***144*** (18)	***153***
Thorax Lateral						
Newborn	6	32 (24)	25 (5)	18 (3)	NA	29
1–5 years	14	44 (85)	44 (46)	42 (27)		44
5–10 years	25	78 (115)	64 (58)	74 (40)		76
10–15 years	44	225 (97)	213 (68)	239 (44)		232
Adult	60	315 (63)	352 (53)	570 (30)		461
Abdomen AP/PA						
Newborn	6	22 (59)	24 (5)	23 (4)	NA	24
1–5 years	14	85 (237)	64 (34)	101 (21)		93
5–10 years	25	***431*** (274)	***417*** (55)	***302*** (28)		***424***
10–15 years	44	***1093*** (315)	***943*** (40)	***1041*** (22)		***1067***
Adult	60	***1554*** (123)	***1275*** (24)	***2812*** (9)		***2183***

**Table 5:** Available National and regional DRLs categorised into mean weights based on WHO and CDC data

MyXrayDose Age Categories	Mean Weight [kg]	DRL [mGy.cm^2^] (Sample size, where available)					
		Europe	Germany	Ireland	Spain (135–1025)	Nordic	Belgium (721)
Pelvis AP/PA							
Newborn	6	NA	NA	39	60	41 (87)	NA
1–5 years	14	NA	150	39	180	41 (87)	
5–10 years	25	180	250	111	310	130 (104)	
10–15 years	44	310	NA	412	400	330 (49)	
Adult	60	NA	NA	800	NA	646 (42)	
Thorax AP/PA							
Newborn	6	22	NA	17	40	40 (128)	20
1–5 years	14	22	25	17	50	40 (128)	35
5–10 years	25	50	35	22	85	28 (137)	50
10–15 years	44	70	NA	50	100	50 (131)	120
Adult	60	87	NA	70	NA	97 (84)	NA
Thorax Lateral							
Newborn	6	NA	NA	NA	NA	NA	40
1–5 years	14		40				70
5–10 years	25		60				100
10–15 years	44		NA				350
Adult	60		NA				NA
Abdomen AP/PA							
Newborn	6	150	NA	63	150	73 (96)	30
1–5 years	14	150	250	63	200	73 (96)	100
5–10 years	25	250	350	100	225	237 (134)	250
10–15 years	44	475	NA	286	300	534 (75)	450
Adult	60	700	NA	457	NA	1540 (28)	NA

**Fig. 1:**
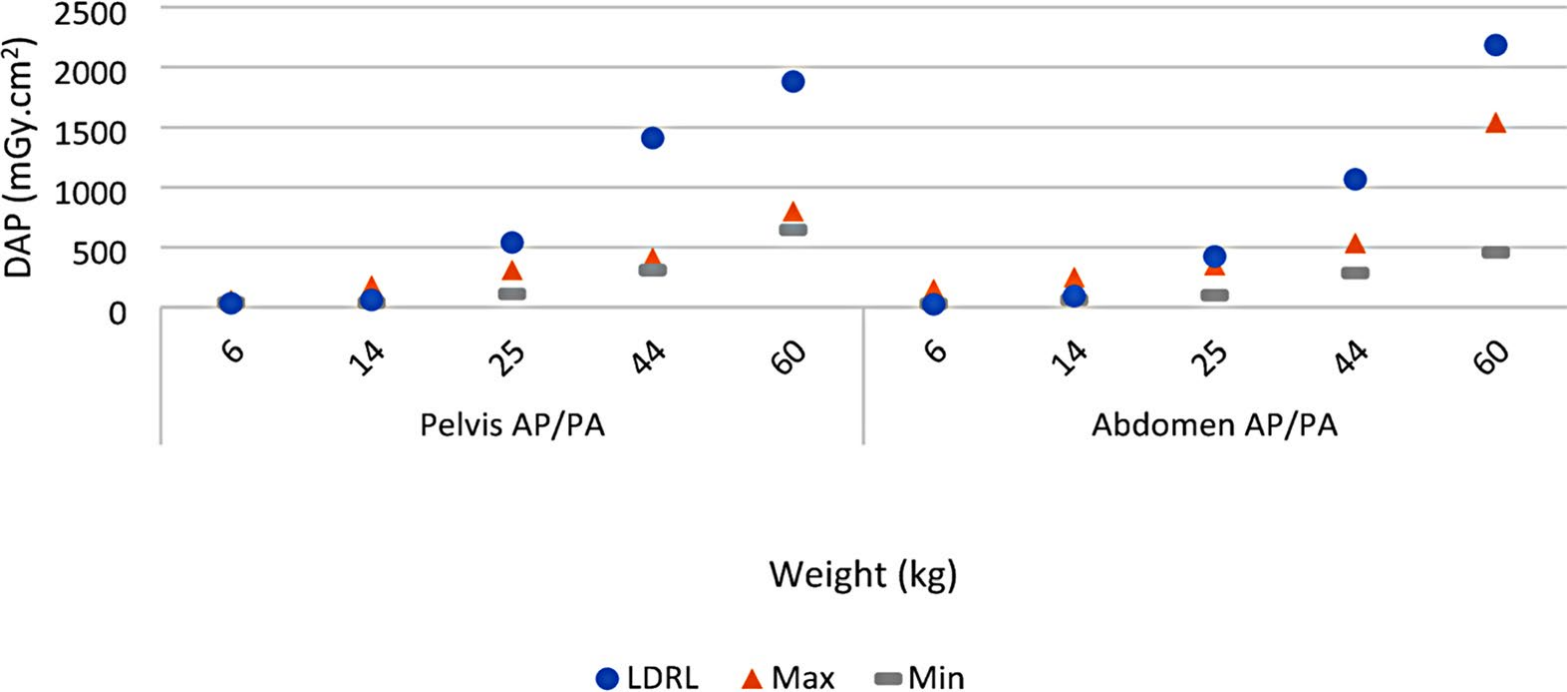
Comparison of RCH LDRL found in this study (Circles), Maximum available published DRL (Triangles), and Minimum available published DRL (Dashes) for Pelvis AP/PA and Abdomen AP/PA protocols

**Fig. 2:**
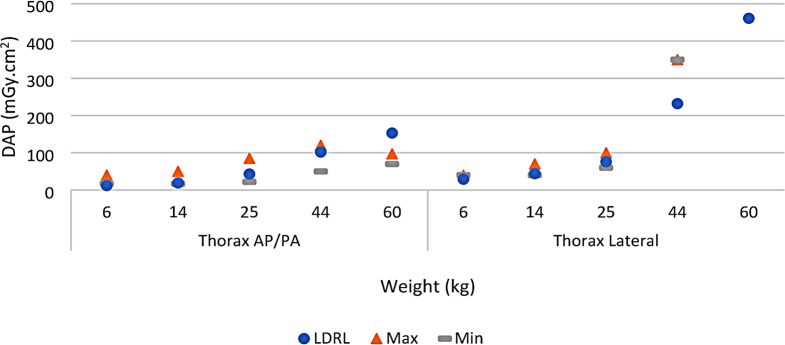
Comparison of RCH LDRL found in this study (Circles), Maximum available published DRL (Triangles), and Minimum available published DRL (Dashes) for Thorax AP/PA and Thorax Lateral protocols

A comparison of RCH LDRLs (from Table 4) and the maximum and minimum available published local, national, and regional DRLs for each protocol (from Table 5) is shown in Fig. 1 (Pelvis AP/PA and Abdomen AP/PA) and Fig. 2 (Thorax AP/PA and Thorax Lateral).

## Discussion

This study compared our facility’s paediatric GXR FRLs to published local, national, and regional DRLs. All of our median DAPs for patients in the 6 kg and 14 kg weight category were below the European or highest available national DRLs for all protocols. This indicates that our doses are in line with international benchmarks for lower weight patients, which comprises of a larger percentage of our patients compared to most generalised hospitals. However, for higher weight patients (greater than 44 kg), the European DRL was exceeded for each examination where it was available (Pelvis AP/PA, Thorax AP/PA, and Abdomen AP/PA). This indicates a need for a review into protocols for patients as they move into adolescence and adulthood. It is also interesting to note that, across all age categories, the dose for thicker anatomy (abdomen and pelvis) recorded at our institute were disproportionality larger relative to the European DRL. On average, our adult thorax exam (145 mGy.cm^2^) is 1.7 times greater than the European DRL (87 mGy.cm^2^) whilst our adult abdomen (1880 mGy.cm^2^) is 2.7 times greater than the European DRL (700 mGy.cm^2^). As aforementioned, DRLs are not limits, and a review involving assessment of image quality is required.

In comparing our institute data to other available DRLs, it is important to identify some limitations. The first is the accuracy of the dose values collected. The IEC-60580 standard [22] requires DAP meter accuracy to within 25%, so accuracy of dose metrics may vary by up to ± 25%. At our institute, the DAP meter accuracy for the fixed units assessed in this study are within 6% at 70 kVp. Despite the known accuracy for our GXR units, the accuracy of other sites to which national DRLs have been derived from is unknown. It is assumed that all data is within the IEC DAP meter accuracy compliance, however this uncertainty is still large and may explain some differences between our institutional data and DRL data.

The use of discrete categories in assessing patient weight can also create limitations due to small patient sample sizes. MyXrayDose categorises patients by age which was then converted to weight, based on WHO and CDC data. With patient cohort sizes being a limitation in the setting of paediatric DRLs, determining methods for maximising the patient data available is important. This limitation is demonstrated by the Pelvis AP/PA examination in Resus for 6 kg patients. MyXrayDose has produced an FRL of 32 mGy.cm^2^ based on a sample of two patients, calling into question the accuracy of this figure. Conversely, our institute completed the Thorax AP/PA protocol 1647 times for a 14 kg patient in Room 1. In this case, when a large sample size is present, it may be worthwhile to create an additional weight category, and in turn and an additional FRL value. By generating more FRLs, a more accurate curve can be derived from institute data.

Data collected through MyXrayDose captures every patient who receives an exposure at our institute, irrespective of complexity or size. This leads to the FRL produced in MyXrayDose being an accurate indicator of the actual dose patients receive at our institute. However, DRL surveys are intended to be completed with a targeted selection toward standard size patients in routine conditions. RCH is the largest paediatric centre in Victoria, leading to an increased level of complexity in patients. For example, RCH receives many high acuity patients with ancillary equipment such as tubes and lines, which can increase the radiation exposure required. Conversely, RCH conducts clinical trials for achondroplasia patients, who may be an older age, but due to atypical bone structure have reduced radiation exposure (i.e., decreased image area).

It is important to consider the technologies that have been assessed in this survey when benchmarking doses. The GXR units and detectors were installed in 2011 at RCH. Table 2 indicates the year of collection for surveys, ranging from 2004 to 2020. During this time, it is possible that some sites were still using computed radiography (CR) systems. Digital Radiography (DR) has improved detector technology and comes with increased detective quantum efficiency (DQE), allowing for a reduction in dose to the patient for a consistent image quality. The available DRLs in literature may present a mixture of DR, CR, and in some cases film. O’Hora et al., in establishing DRLs in Ireland included in their collection survey an option for participating facilities to include what detector technology was used. In cases where DRLs using different detector types were deemed to be statistically significant, equipment-specific DRLs were set [19]. However, for all available data, only one national DRL was set, combining all available detector technologies. Therefore, although this study was performed on DR, it is being compared to DRLs that could be made up of older technologies.

DRL surveys completed should also record the technical parameters used at each site, such as the use of grids, automatic-exposure-control (AEC) and detector system, as these have an impact on radiation dose. At our institute, grids are used for Pelvis and Abdomen examinations for patients greater than 25 kg, and for Thorax examinations for patients greater than 70 kg. As technical parameters may vary from site to site, including this information may be of value. In this circumstance when our site’s FRLs are exceeding the European DRL, being provided with this information would allow us to investigate potential optimisation pathways.

Based on this study, it is evident that protocol optimisation needs to be performed on larger patients at our institute. Protocol optimisation should not just consider dosimetry from DRLs alone but must also consider image quality. Therefore, the next step at our institute is to complete a multi-disciplinary image quality assessment to identify if it is possible to reduce the radiation dose.

Despite the limitations above, this study begins to create the conversation for the establishment of paediatric GXR DRLs in an Australian context. Whilst there is currently no Australian paediatric GXR DRLs, this study may prove a useful benchmarking tool for other local Australian sites to compare to. This study highlights the need for future work, in potentially running an Australian DRL survey in both paediatric and non-paediatric specific centres to formulate paediatric GXR DRLs. This survey data can then be used to generate DRL curves, like that in Almen et al., and allow for other facilities to benchmark their GXR doses. A major benefit of said DRL curve allows for facilities with small patient cohorts to assess their doses as a function of the patient weight, allowing for optimisation despite the small number of examinations. A DRL curve will also remove the reliance on WHO and CDC health data to map age to weight and be a more accurate reflection of patient cohorts in Australia.

Additionally, this study assessed commonly performed examinations to which there were available local, national, and regional DRLs. Future work may expand to include other protocols, such as a Femur AP. No national DRLs could be located for this, however at our institute we conducted 674 total examinations during the period, in which an FRL for 60 kg patients was determined to be 367 mGy.cm^2^ (Room 1).

## Conclusion

DRLs provide an indication of the amount of radiation used in routine conditions for a specific protocol. This study compared the DAP from fixed GXR units at our institute, to published international DRLs. The results demonstrate that small patients are within available DRLs, whilst the DAP to higher weight patients are exceeding DRLs. Whilst DRLs are not regulatory limits, the study highlights the need for optimisation of some GXR protocols at our institute. However, the data provided in this study, allows other local sites within Australia to have a local benchmark available. Future research may include a collection of Australian sites to attempt to benchmark doses nationally.
